# Supplementary material for: Resolving the full spectrum of human genome variation using Linked-Reads
Source: Genome Res. 2019 Apr;29(4):635–45. doi: 10.1101/gr.234443.118 (PMC6442396; doi:10.1101/gr.234443.118)
Supplement: Supplemental Material [file supp_gr.234443.118_Supplemental_longranger-2.2.zip › longranger-2.2.tar.bz2!/lr2.2-public/lib/stan/cmdstan/stan/doc/api/html/agrad_2rev_2functions_2lgamma_8hpp_source.html]

Stan: src/stan/agrad/rev/functions/lgamma.hpp Source File


|  |  |
| --- | --- |
|  | Stan  2.5.0  probability, sampling & optimization |


- Main Page
- Related Pages
- Namespaces
- Classes
- Files

- File List
- File Members

All Classes Namespaces Files Functions Variables Typedefs Enumerations Enumerator Friends Macros Pages

- src
- stan
- agrad
- rev
- functions

lgamma.hpp

Go to the documentation of this file.

1 #ifndef STAN\_\_AGRAD\_\_REV\_\_FUNCTIONS\_\_LGAMMA\_HPP

2 #define STAN\_\_AGRAD\_\_REV\_\_FUNCTIONS\_\_LGAMMA\_HPP

3

4 #include <valarray>

5 #include <stan/agrad/rev/var.hpp>

6 #include <stan/agrad/rev/internal/v\_vari.hpp>

7 #include <stan/math/constants.hpp>

8 #include <boost/math/special\_functions/digamma.hpp>

9 #include <boost/math/special\_functions/gamma.hpp>

10

11 namespace stan {

12  namespace agrad {

13

14  namespace {

15  class lgamma\_vari : public op\_v\_vari {

16  public:

17  lgamma\_vari(double value, vari\* avi) :

18  op\_v\_vari(value, avi) {

19  }

20  void chain() {

21  avi\_->adj\_ += adj\_ \* boost::math::digamma(avi\_->val\_);

22  }

23  };

24  }

25

36  inline var lgamma(const stan::agrad::var& a) {

37  double lgamma\_a = boost::math::lgamma(a.val());

38  return var(new lgamma\_vari(lgamma\_a, a.vi\_));

39  }

40

41  }

42 }

43 #endif

stan::agrad::var::val

double val() const

Return the value of this variable.

**Definition:** var.hpp:209

stan::agrad::lgamma

fvar< T > lgamma(const fvar< T > &x)

**Definition:** lgamma.hpp:15

stan::agrad::var::vi\_

vari \* vi\_

Pointer to the implementation of this variable.

**Definition:** var.hpp:40

v\_vari.hpp

stan::math::lgamma

double lgamma(double x)

**Definition:** lgamma.hpp:31

var.hpp

stan::agrad::var

Independent (input) and dependent (output) variables for gradients.

**Definition:** var.hpp:27

stan::agrad::digamma

fvar< T > digamma(const fvar< T > &x)

**Definition:** digamma.hpp:16

constants.hpp


---

[ Stan Home Page ]

*© 2011–2014,
Stan Development Team.*
